# Considerations for Balance Between Fundamental Treatment and Improvement of Quality of Life of Pediatric Thyroid Cancer Patient: Comparative Analysis With Adult Using Propensity Score Matching

**DOI:** 10.3389/fped.2022.840432

**Published:** 2022-04-28

**Authors:** Ji Young You, Se-Woong An, Hoon Yub Kim, Da Won Park, Hyung Kwon Byeon, Serena Patroniti, Gianlorenzo Dionigi, Ralph P. Tufano

**Affiliations:** ^1^Department of Surgery, KUMC Thyroid Center, Korea University Hospital, Korea University College of Medicine, Seoul, South Korea; ^2^Department of Otorhinolaryngology – Head and Neck Surgery, Soonchunhyang University College of Medicine, Seoul, South Korea; ^3^Division of Pediatrics, University of Messina, Messina, Italy; ^4^Division of General Surgery, Endocrine Surgery Section, Istituto Auxologico Italiano IRCCS (Istituto di ricovero e cura a carattere scientifico), Milan, Italy; ^5^Department of Pathophysiology and Transplantation, University of Milan, Milan, Italy; ^6^Department of Otolaryngology – Head and Neck Surgery, The Johns Hopkins University School of Medicine, Baltimore, MD, United States

**Keywords:** pediatric cancer, thyroid malignancy, total thyroidectomy, thyroid lobectomy, propensity score matching

## Abstract

**Background:**

Thyroid cancer is very rarely observed in children and adolescents, some reports have shown that the long-term outcome of treatment is better than that of adult patients, despite many treatment failures or a high risk of recurrence. This study considers whether it is appropriate to treat pediatric thyroid cancer patients aggressively, as per the ATA guidelines, based on the balance between the fundamental treatment of thyroid cancer and the improvement of the long-term quality of life of pediatric patients.

**Methods:**

A total of 1,950 patients were recruited, including 83 pediatric and 1,867 adult patients, who were diagnosed with thyroid cancer and underwent surgical treatment at one of our medical center hospitals from March 2000 to January 2020.

**Results:**

Sixty-nine pairs of pediatric and adult patients were matched in a ratio of 1:2 through propensity score matching. When compared through propensity score matching, there was no significant difference in prognosis such as recurrence rate in children and adults at the same stage.

**Conclusion:**

This study showed that the prognosis of both pediatric and adult patients who underwent a total thyroidectomy and lobectomy was not significantly different. If more pediatric patients can be considered for the less-aggressive lobectomy than a total thyroidectomy through various preoperative examinations and meticulous pre-diagnosis, it may be possible to properly determine the balance between improving long-term quality of life while providing fundamental cancer treatment.

## Introduction

Thyroid cancer is very rarely observed in children and adolescents, and it is reported that only approximately 1.9% of new thyroid cancer patients develop it in childhood annually (1, 2). However, papillary thyroid carcinoma, which is the most common type of thyroid cancer, accounts for 90–95% of pediatric thyroid cancer; other types are reported to be very rare (3–5).

Because childhood thyroid cancer has a more extensive invasion and a higher recurrence rate than adult thyroid cancer, aggressive treatment for differentiated thyroid cancer in children is recommended, i.e., a thyroidectomy is recommended over a lobectomy (4, 6, 7). Thyroid cancer in pediatric patients is generally treated according to the American Thyroid Association (ATA) pediatric thyroid cancer guidelines, which are primarily based on Mayo clinic data from patients in the United States (8, 9). This is because very few cases are detected through screening, and most cases are in an advanced state at the time of diagnosis.

However, some reports have shown that the long-term outcome of treatment is better than that of adult patients, despite many treatment failures or a high risk of recurrence (10, 11). Compared with a lobectomy, a total thyroidectomy has a higher risk of complications, such as hypothyroidism and recurrent laryngeal nerve damage after surgery. Therefore, pediatric patients suffer from sequelae for a longer period than adults, and they are more likely to experience a reduced quality of life (12). A South Korean research team reported that a lobectomy alone is sufficient for the treatment of early cancer in pediatric patients (13). South Korea is an iodine-rich country; therefore, thyroid function tests are frequently performed, even on children. Thus, there are many cases in which thyroid cancer is diagnosed at an early stage through tests, such as ultrasound (14). At the pediatric endocrine clinic, when testing growth hormone in children, a thyroid function test is also performed. In addition, it is estimated that thyroid cancer is frequently diagnosed due to the high incidence of thyroid cancer and good access to clinics that perform thyroid ultrasound.

This study considers whether it is appropriate to treat pediatric thyroid cancer patients aggressively, as per the ATA guidelines, based on the balance between the fundamental treatment of thyroid cancer and the improvement of the long-term quality of life of pediatric patients.

## Materials and Methods

### Patients

**FIGURE 1:**
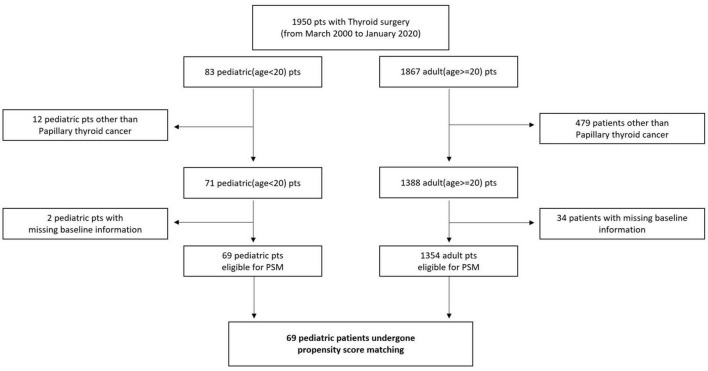
Study design flowchart.

Patients who were diagnosed with thyroid cancer and underwent surgical treatment at one of three hospitals (Anam, Guro, and Ansan) of the Korea University Medical Center from March 2000 to January 2020 were recruited. Patients who underwent surgery from the Head and Neck Department of Otolaryngology or Endocrine Department of General Surgery were also included. A total of 1,950 patients were recruited, including 83 pediatric and 1,867 adult patients. According to the standard definition, children were defined as those aged younger than 20 years. Twelve pediatric patients and 479 adult patients did not have papillary thyroid cancer (PTC) based on a pathological examination, and they were excluded from the study. Additionally, two pediatric and 34 adult patients did not have baseline information (tumor size); thus, they did not have a propensity score for the propensity score matching and were also excluded (Figure 1). All patients were enrolled retrospectively, and clinical data were analyzed *via* chart review. This retrospective study was approved by the Institutional Review Board of the Korea University College of Medicine.

### Surgical Strategy

Pediatric PTC patients were treated according to the American Thyroid Association guidelines. The guidelines recommend a total thyroidectomy because lesions are often bilateral and multifocal in pediatric patients. However, in this study, those patients who only had intrathyroidal lesions and no bilaterality underwent a lobectomy. Prophylactic central lymph node dissection was performed in all pediatric patients. A therapeutic modified radical neck dissection (MRND) was performed when N1b disease was suspected or pathologically confirmed.

Adult PTC patients were treated according to the National Comprehensive Cancer Network (NCCN) guidelines. Patients who satisfied all the following conditions underwent a lobectomy instead of a total thyroidectomy: (I) unilateral disease, (II) primary tumor size < 4 cm, (III) no extrathyroidal extension (ETE) or lympho-vascular invasion, and (IV) no clinically suspicious lymph nodes in the lateral neck. Therapeutic central and lateral neck dissections were performed on patients who were clinically suspected of having neck node metastasis.

Because the surgeons who performed thyroid cancer surgeries for pediatric and adult patients in this study had more than 10 years of experience, we assumed that there was no difference in the skill level of the surgeons and no respective bias.

Open thyroidectomies were performed on most pediatric and adult patients. Twelve adult patients underwent endoscopic thyroidectomies (9, lobectomy; 3, total), and 13 pediatric patients underwent robotic or endoscopic thyroidectomies (5, retro-auricular endoscopic lobectomy; 3, retro-auricular robotic lobectomy; 5, transoral robotic thyroidectomy). There was no significant difference in the surgical approach and method between the two patient groups.

### Postoperative Treatment and Follow Up

All patients received a suppressive dose of levothyroxine after surgery and were regularly followed up through physical examinations, thyroid function tests, and neck ultrasonograms. Initially, the patients were followed up at 3 and 6 months after surgery. Subsequently, they were followed up once every year. When metastasis to the neck node or recurrence in the remaining thyroid tissues were suspected, fine needle aspiration cytology (FNA) was performed for confirmation. Computed tomography (CT), positron emission tomography (PET), and ^131^iodine whole-body scans were also performed to assess the recurrence of PTC when necessary.

### Clinicopathological Variables

The following clinicopathological variables were assessed: age, sex, body mass index (BMI), preoperative FNA cytology, type of surgery, radioactive iodine (RAI) therapy, recurrence, follow-up duration, bilaterality, multiplicity, tumor size, ETE, total harvested lymph nodes (LNs), central LNs, lateral LNs, total positive LNs, positive central LNs, positive lateral LNs, pT stage, and pN stage.

The tumor-node-metastasis (TNM) stage was classified based on the 8th edition of the American Joint Committee on Cancer (AJCC)/Union for International Cancer Control (UICC) TNM staging system.

The 8th AJCC guideline was newly released in 2018; therefore, the staging of thyroid cancer changed from the 7th guideline. Thus, the pathologic reports of patients who underwent surgical treatment before 2018 were reviewed for new staging classifications. If ETE to the strap muscle was not indicated in the pathologic reports, staging was re-classified according to the tumor size, even for pT3 tumors. For example, extension to the perithyroidal soft tissue or minimal extrathyroidal extension was considered to be tumors below pT3a, and the pT stage was determined based only on the size of the tumor.

Patients who underwent unilateral and bilateral surgeries were grouped into the lobectomy and total thyroidectomy categories, respectively. If the staging operation was planned from the initial operation, and a completion thyroidectomy was performed, the patient was included in the total thyroidectomy category. Patients who underwent a lobectomy at a different institution and a completion thyroidectomy at our institution, owing to tumor recurrence, were excluded from the study because the surgeon who conducted the initial operation was different (13 patients).

### Statistical Analysis

Statistical analysis was performed using R (R version 4.0.3 for Windows, R Core Team) (15, 16).

Clinicopathological factors of pediatric and adult PTC patients were compared. Continuous variables were expressed as mean and standard deviation (SD) values, and categorical variables were presented as numeric values and percentages. For a comparison of continuous variables, a normal distribution was first confirmed using the Shapiro–Wilk test. *T*-tests and Wilcoxon rank sum tests were then performed to compare continuous variables that were and were not normally distributed, respectively. For categorical variables, the chi-square test was performed, and Fisher’s exact test was performed when errors occurred (“moonBook” package of R was used for the analysis).

In this study, differences in the prognosis of pediatric and adult patients according to the surgical method were assessed. However, this was a retrospective cohort study, and the number of pediatric patients (71) was too low. Therefore, to reduce the effects of selection bias and confounding variables, adult patients were matched with pediatric patients using propensity score matching. The number of adult patients (1,388) was greater than that of the pediatric patients; thus, one-to-many matching was performed. One-to-two matching was conducted according to previous studies that recommended one-to-one or one-to-two matching (17).

A logistic regression model was used to calculate the propensity scores of the patients. The following prognostic factors were included: sex, tumor size group, ETE, and pN.

Initially, sex, pT, and pN were evaluated to calculate the propensity scores of the patients. However, the pT stage in our data was reported using the previous staging system, AJCC 7th edition. Therefore, to avoid matching errors, the pT was recalculated based on the tumor size and ETE. The tumor size was divided into three groups based on the criteria of the new AJCC 8th edition T staging of tumors exceeding 1 and 2 cm in size. Tumors that were greater than 4 cm in size were rare; thus, they were excluded. Therefore, the propensity score was determined based on sex, tumor size group, ETE, and pN (the “matchit” package of R was used for the analysis) (18, 19).

The Kaplan-Meier survival analysis was performed to plot survival curves, and the log-rank test was conducted to compare propensity-matched patients. A *p*-value < 0.05 was considered statistically significant (‘survival’ package and “survminer” package of R were used for this analysis).

## Results

### Comparison of Baseline Clinicopathologic Characteristics According to Age Group, Before Propensity Score Matching

The baseline clinicopathological characteristics of 71 pediatric and 1,388 adult patients who underwent thyroid surgery for the treatment of PTC are shown in Table 1.

**TABLE 1:** Clinico-pathologic characteristics of patients according to age group.

	Age < 20 (*N* = 71)	Age ≥ 20 (*N* = 1388)	*P*-value
Sex			0.060
Male	9 (12.7%)	319 (23.0%)	
Female	62 (87.3%)	1069 (77.0%)	
Age (years)	16.6 ± 2.8 (15.5–18.5)	51.2 ± 12.4 (42.0–60.0)	<0.05
Body mass index (BMI)	22.4 ± 3.2 (19.9–24.1)	24.9 ± 3.8 (22.3–26.9)	<0.05
Preoperative FNA cytology (Bethesda classification)			0.037
I	2 (3.1%)	6 (0.4%)	
II	2 (3.1%)	15 (1.1%)	
III	6 (9.2%)	70 (5.1%)	
IV	1 (1.5%)	22 (1.6%)	
V	12 (18.5%)	322 (23.3%)	
VI	42 (64.6%)	945 (68.5%)	
Surgery type			0.803
Lobectomy	19 (26.8%)	342 (24.7%)	
Right	14 (19.7%)	166 (12.0%)	
Left	5 (7.0%)	176 (12.7%)	
Total thyroidectomy	52 (73.2%)	1042 (75.3%)	
Radioactive iodine (RAI)			0.274
Yes	41 (59.4%)	687 (66.6%)	
No	28 (40.6%)	344 (33.4%)	
Recurrence			<0.05
Yes	11 (15.5%)	43 (3.1%)	
No	60 (84.5%)	1345 (96.9%)	
Follow up duration (months)	89.9 ± 57.5 (43.5–126.0)	77.5 ± 36.5 (48.0–104.0)	0.192
Bilaterality			<0.05
Yes	4 (5.7%)	376 (27.6%)	
No	66 (94.3%)	986 (72.4%)	
Multiplicity			0.001
Yes	15 (21.1%)	570 (41.2%)	
No	56 (78.9%)	813 (58.8%)	
Tumor size (cm)	2.1 ± 1.6 (0.9–2.8)	0.9 ± 0.8 (0.5–1.1)	<0.05
Tumor size in group			<0.05
Size ≤ 1cm	21 (30.0%)	1031 (74.4%)	
1 cm < Size ≤ 2 cm	20 (28.6%)	274 (19.8%)	
Size > 2 cm	29 (41.4%)	81 (5.8%)	
Extrathyroidal extension (ETE)			0.007
Yes	42 (59.2%)	585 (42.2%)	
No	29 (40.8%)	800 (57.8%)	
Total harvested LNs	20.3 ± 25.8	11.0 ± 10.2	0.801
Central LNs	9.9 ± 8.5	9.6 ± 8.1	0.856
Lateral LNs	12.6 ± 23.4	1.4 ± 6.0	<0.05
Total positive LNs	7.4 ± 8.0	2.0 ± 4.2	<0.05
Positive central LNs	5.3 ± 5.5	1.7 ± 3.5	<0.05
Positive lateral LNs	2.8 ± 5.2	0.3 ± 1.7	<0.05
pTstage			<0.05
T1a	22 (31.0%)	1029 (74.1%)	
T1b	19 (26.8%)	271 (19.5%)	
T2	21 (29.6%)	67 (4.8%)	
T3a	7 (9.9%)	9 (0.6%)	
T3b	2 (2.8%)	8 (0.6%)	
T4a	0 (0.0%)	4 (0.3%)	
T4b	0 (0.0%)	0 (0.0%)	
pN stage			<0.05
N0	30 (42.3%)	777 (56.0%)	
N1a	27 (38.0%)	528 (38.0%)	
N1b	14 (19.7%)	83 (6.0%)	

*Data are expressed as the patient number (%) or mean ± SD.*

The mean age of the pediatric and adult patients was 16.6 ± 2.8 and 51.2 ± 12.4 years, respectively (*p* < 0.05). Because the prevalence of thyroid cancer in pre-pubertal and post-pubertal children is different, this should be considered. There was a significant BMI difference between pediatric and adult patients (22.4 ± 3.2, 24.9 ± 3.8, respectively, *p* < 0.05). Nineteen (26.8%) pediatric patients and 342 (24.7%) adult patients underwent a lobectomy, whereas 52 (73.2%) pediatric patients and 1042 (75.3%) adult patients underwent a total thyroidectomy. There was no significant difference in the surgical method between the two patient groups (*p* = 0.803). A recurrence of PTC was observed in 11 (15.5%) pediatric and 43 (3.1%) adult patients. The number of recurrence cases differed significantly between the two groups (*p* < 0.05). The mean follow-up durations for pediatric and adult patients were 89.9 ± 57.5 and 77.5 ± 36.5, respectively, and there was no significant difference between the two groups (*p* = 0.192). Bilaterality was observed in 4 (5.7%) pediatric and 376 (27.6%) patients, which was significantly different between the two groups (*p* = 0.000). Additionally, the number of multiplicity cases was 15 (21.1%) and 570 (41.2%) in pediatric and adult patients, respectively, which was significantly different between the two groups (*p* = 0.001). The tumor size was 2.1 ± 1.6 cm and 0.9 ± 0.8 cm in pediatric and adult patients, respectively, which was significantly different between the two groups (*p* < 0.05). Forty-two (59.2%) pediatric and 585 (42.2%) adult patients had ETE, which indicates that ETE was significantly more common in adult patients (*p* = 0.007). The number of total harvested LNs was 20.3 ± 25.8 in pediatric patients and 11.0 ± 10.2 in adult patients, which was not different between the two groups (*p* = 0.801). In contrast, the number of total positive LNs was 7.4 ± 8.0 and 2.0 ± 4.2 in pediatric and adult patients, respectively, which was significantly different between the two groups (*p* < 0.05). The distribution of pT stages in pediatric and adult patients were as follows (pediatric and adult patients, respectively): pT1a, 22 (31.0%) and 1029 (74.1%); pT1b, 19 (26.8%) and 271 (19.5%); pT2, 21 (29.6%) and 67 (4.8%); pT3a, 7 (9.9%) and 9 (0.6%); pT3b, 2 (2.8%) and 8 (0.6%); pT4a, 0 (0.0%) and 4 (0.3%); pT4b, 0 (0.0%) and 0 (0.0%). There were significant differences in the pT stages between the two patient groups (*p* < 0.05). The distribution of pN stages in pediatric and adult patients were as follows (pediatric and adult patients, respectively): pN0, 30 (42.3%) and 777 (56.0%); pN1a, 27 (38.0%) and 528 (38.0%); pN1b, 14 (19.7%) and 83 (6.0%). There were significant differences in the pN stages between the two patient groups (*p* < 0.05).

### Comparison of Clinicopathologic Characteristics of Propensity-Matched Patients According to Age Group

Sixty-nine pairs of pediatric and adult patients were matched in a ratio of 1:2 through propensity score matching. The clinicopathologic characteristics of the matched patients are shown in Table 2. The mean age of the pediatric and adult patients was 16.8 ± 2.4 and 38.4 ± 14.0 years, respectively (*p* < 0.05). Eighteen (26.1%) pediatric and 25 (18.1%) adult patients underwent a lobectomy, and 51 (73.9%) pediatric and 113 (81.9%) adult patients underwent a total thyroidectomy. There was no significant difference in the surgical method between the two patient groups (*p* = 0.250). Unlike the comparison results of the two groups before matching, the tumor size was 2.1 ± 1.6 cm and 1.8 ± 1.5 cm in pediatric and adult patients, respectively, which was not significantly different (*p* = 0.185).

**TABLE 2:** Clinico-pathologic characteristics of propensity-matched PTC patients according to age group.

	Age < 20 (*N* = 69)	Age ≥ 20 (*N* = 138)	*P*-value
Sex			NA
Male	9 (13.0%)	18 (13.0%)	
Female	60 (87.0%)	120 (87.0%)	
Age (years)	16.8 ± 2.4 (16–19)	38.4 ± 14.0 (29–41)	<0.05
Body Mass Index (BMI)	22.5 ± 3.3 (19.9–24.2)	24.2 ± 4.5 (20.9–27.5)	0.009
Surgery type			0.250
Lobectomy	18 (26.1%)	25 (18.1%)	
Total thyroidectomy	51 (73.9%)	113 (81.9%)	
Bilaterality			0.012
Yes	4 (5.8%)	28 (20.3%)	
No	65 (94.2%)	110 (79.7%)	
Multiplicity			0.018
Yes	14 (20.3%)	52 (37.7%)	
No	55 (79.7%)	86 (62.3%)	
Tumor size (cm)	2.1 ± 1.6	1.8 ± 1.5	0.185
Tumor size in group			NA
≤1 cm	20 (29.0%)	40 (29.0%)	
>1 cm and ≤2 cm	20 (29.0%)	40 (29.0%)	
>2 cm	29 (42.0%)	58 (42.0%)	
Extrathyroidal extension (ETE)			0.960
Yes	41 (59.4%)	84 (60.9%)	
No	28 (40.6%)	54 (39.1%)	
pT stage			0.059
T1a	20 (29.0%)	40 (29.0%)	
T1b	19 (27.5%)	40 (29.0%)	
T2	21 (30.4%)	51 (37.0%)	
T3a	7 (10.1%)	4 (2.9%)	
T3b	2 (2.9%)	0 (0.0%)	
T4a	0 (0.0%)	3 (2.2%)	
pN stage			0.368
N0	28 (40.6%)	51 (37.0%)	
N1a	27 (39.1%)	67 (48.6%)	
N1b	14 (20.3%)	20 (14.5%)	
Recurrence			0.184
Yes	11 (15.9%)	12 (8.7%)	
No	58 (84.1%)	126 (91.3%)	

*Data are expressed as the patient number (%) or mean ± SD.*

Extrathyroidal extension was observed in 41 (59.4%) pediatric and 84 (60.9%) adult patients, indicating that ETE was not significantly different (*p* = 0.960). The distribution of pT stages in pediatric and adult patients were as follows (pediatric and adult patients, respectively): pT1a, 20 (29.0%) and 40 (29.0%); pT1b, 19 (27.5%) and 40 (29.0%); pT2, 21 (30.4%) and 51 (37.0%); pT3a, 7 (10.1%) and 4 (2.9%); pT3b, 2 (2.9%) and 0 (0.0%); pT4a, 0 (0.0%) and 3 (2.2%); pT4b, 0 (0.0%) and 0 (0.0%). There were no significant differences in the pT stages between the two patient groups (*p* = 0.059). The distribution of pN stages in pediatric and adult patients were as follows (pediatric and adult patients, respectively): pN0, 28 (40.6%) and 51 (37.0%); pN1a, 27 (39.1%) and 67 (48.6%); pN1b, 14 (20.3%) and 20 (14.5%). There were no significant differences in the pT stages between the two patient groups (*p* = 0.386). Recurrence was observed in 11 (15.5%) pediatric and 12 (8.7%) adult patients after surgery. The number of recurrent cases was not significantly different between the two groups (*p* = 0.184).

### Disease Free Survival Analysis of Propensity-Matched Patients According to Age Group

**FIGURE 2:**
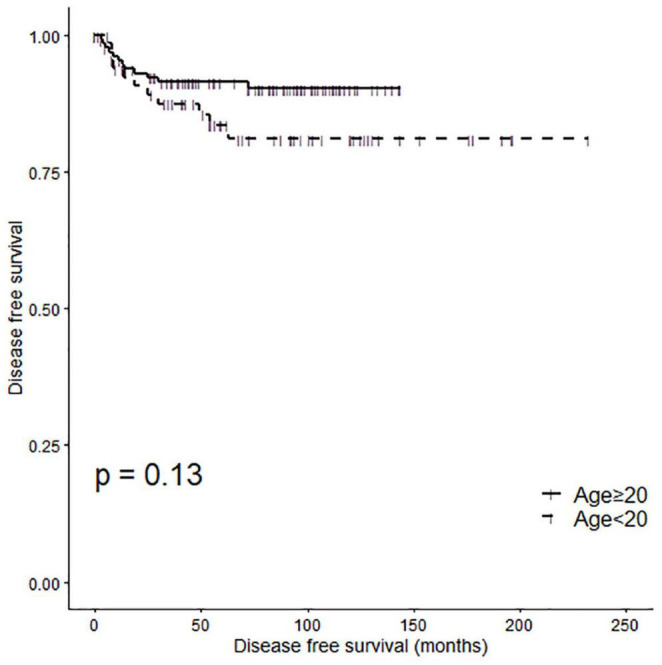
Disease-free survival rates of subgroups according to age group (log rank *p* = 0.13).

A Kaplan-Meier analysis demonstrated no significant difference in disease-free survival (DFS) between the two patient groups (Figure 2, log-rank *P* = 0.13). The 5 Year disease free survival rate (5Y DFSR) was 83.54% (lower 95% CI: 74.62%, upper 95% CI: 93.52%) and 91.46% (lower 95% CI: 86.75%, upper 95% CI: 96.42%) in pediatric and adult patients, respectively.

**FIGURE 3:**
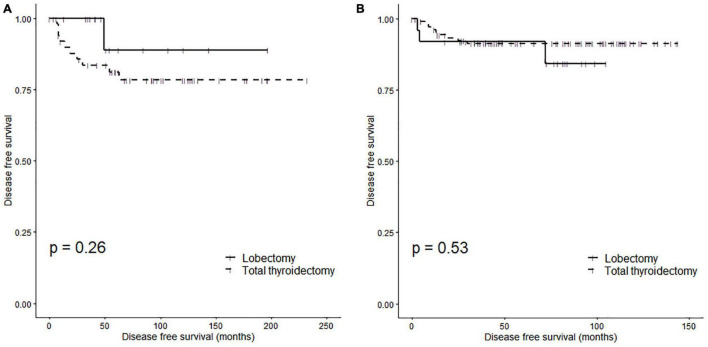
Disease-free survival rates of subgroups according to surgery type: **(A)** pediatric patients (log rank *p* = 0.26) and **(B)** adult patients (log rank *p* = 0.53).

Figure 3 shows the Kaplan-Meier curve of the DFS according to the surgical method in pediatric (Figure 3A) and adult (Figure 3B) patients. There was no significant difference in DFS according to the surgical method in pediatric patients (log-rank *p* = 0.26). The 5Y DFSR was 88.90% (lower 95% CI: 70.60%, upper 95% CI: 100.00%) and 81.16% (lower 95% CI: 70.75%, upper 95% CI: 93.10%) in pediatric patients who underwent a lobectomy and total thyroidectomy, respectively. Similarly, there was no significant difference in DFS according to the surgical method in adult patients (log-rank *p* = 0.53). The 5Y DFSR was 92.00% (lower 95% CI: 81.96%, upper 95% CI: 100.00%) and 91.32% (lower 95% CI: 86.05%, upper 95% CI: 96.91%) in adult patients who underwent a lobectomy and total thyroidectomy, respectively.

## Discussion

From the results of our study, it can be concluded that the prognosis was sufficiently good, even in pediatric patients and with standard treatment according to the stage of cancer, such as tumor size, ETE, and pN stage. The detection and diagnosis of cancer tends to be delayed in pediatric patients compared to adult patients. However, if detected early, a lobectomy can be performed, and recurrence and metastasis can be prevented and treated early through continuous follow-up. This treatment approach can considerably improve the quality of life of pediatric patients with thyroid cancer in the future. In South Korea, patients have excellent access to hospitals, owing to the universal supply of the national health insurance system. Additionally, owing to the high coverage of private insurance, it is not uncommon for young patients to undergo thyroid ultrasound examinations and fine needle aspiration examinations (20). Thus, an environment that is favorable for the early detection of thyroid cancer has been established.

A total thyroidectomy can fundamentally reduce the recurrence of thyroid cancer by removing the entire thyroid tissue; however, it can cause various complications and sequelae in pediatric patients who require long-term follow-up. A total thyroidectomy is known to have a higher risk of complications, such as hypothyroidism and recurrent laryngeal nerve damage after surgery, than a lobectomy (21). Patients may also have to take calcium supplements throughout their lives, owing to hypoparathyroidism. Moreover, patients who undergo a total thyroidectomy must take levothyroxine for the rest of their lives. In some patients, the accumulation of free T4 from levothyroxine increases the burden on the heart, which may cause heart failure (22). There are also reports that long-term use of levothyroxine may promote osteoporosis in postmenopausal women (23, 24). Therefore, it is necessary to actively recommend a lobectomy for the treatment of pediatric thyroid cancer patients who do not require a total thyroidectomy.

## Conclusion

This study showed that the prognosis of both pediatric and adult patients who underwent a total thyroidectomy and lobectomy was not significantly different. If more pediatric patients can be considered for the less-aggressive lobectomy than a total thyroidectomy through various preoperative examinations and meticulous pre-diagnosis, it may be possible to properly determine the balance between improving long-term quality of life while providing fundamental cancer treatment.

## Data Availability Statement

The original contributions presented in the study are included in the article/supplementary material, further inquiries can be directed to the corresponding author.

## Ethics Statement

This retrospective study was reviewed and approved by the Institutional Review Board of the Korea University College of Medicine.

## Author Contributions

JY, S-WA, HB, DP, and HK: substantial contributions to the conception or design of the work, acquisition, analysis, or interpretation of data for the work. JY and S-WA: drafting the work or revising it critically for important intellectual content. GD, RT, SP, and HK: final approval of the version to be published. JY, S-WA, HK, DP, HB, SP, GD, and RT: agreement to be accountable for all aspects of the work in ensuring that questions related to the accuracy or integrity of any part of the work are appropriately investigated and resolved. All authors contributed to the article and approved the submitted version.

## Conflict of Interest

The authors declare that the research was conducted in the absence of any commercial or financial relationships that could be construed as a potential conflict of interest.

## Publisher’s Note

All claims expressed in this article are solely those of the authors and do not necessarily represent those of their affiliated organizations, or those of the publisher, the editors and the reviewers. Any product that may be evaluated in this article, or claim that may be made by its manufacturer, is not guaranteed or endorsed by the publisher.
